# Small Bowel Angioedema Secondary to Angiotensin-Converting Enzyme Inhibitors

**DOI:** 10.7759/cureus.943

**Published:** 2016-12-26

**Authors:** Faisal Inayat, Abu Hurairah

**Affiliations:** 1 Department of Medicine, New York-Presbyterian Hospital, Weill Cornell Medical College, New York City, NY, USA; 2 Division of Gastroenterology, Department of Medicine, SUNY Downstate Medical Center, Brooklyn, NY, USA

**Keywords:** ace inhibitor, angioedema, small bowel edema, adverse effect, correct diagnosis, awareness

## Abstract

Small bowel angioedema induced by angiotensin-converting enzyme (ACE) inhibitors is a rare clinicopathologic entity. It frequently poses a diagnostic challenge and is often not recognized before surgical exploration. The present study illustrates that clinical awareness for this condition and adequate use of radiologic investigations can help make the correct diagnosis of ACE inhibitor-associated angioedema, thus avoiding the cost and morbidity associated with unnecessary interventions.

## Introduction

Angioedema is an infrequent adverse effect associated with the use of ACE inhibitors. Approximately 0.1-0.7% of patients taking ACE inhibitors develop angioedema, and it frequently affects the face, particularly the mucous membranes of the oropharynx, and upper airway [1-2]. However, ACE inhibitor-induced small bowel angioedema is rare and its incidence is not well described [2]. These patients usually present with nonspecific symptoms such as abdominal pain, nausea, vomiting, and diarrhea [1-2]. On the radiologic assessment of the abdomen, free fluid is often present with small bowel edema [3]. Laboratory evaluation often demonstrates an elevated white cell count [3]. A detailed clinical history, including active medications, is the key to diagnosing patients with ACE inhibitor-related small bowel angioedema.

## Case presentation

A 53-year-old woman presented to our institution with abdominal pain and diarrhea for one day. The pain was severe, intermittent, crampy, and localized to the left lower quadrant with no alleviating or aggravating factors. The patient reported nonbloody, watery diarrhea without mucous. She had a bowel movement every two to three hours immediately after returning from a four-week stay in Tibet, China. However, no clear association of pain with a specific type of food was identified. The patient denied fever, chills, dyspnea, chest pain, dysuria, hematuria, and sick contacts. She was a drug-free, non-alcoholic non-smoker and did not have any drug or food allergies. Her medical history was significant for hypertension. It was effectively treated with lisinopril (Prinivil ®, Merck) 20 mg/day, which she had been on for two months. On admission, the patient had normal vital signs. Abdominal examination was concerning for significant epigastric tenderness in the right upper quadrant and left lower quadrant. Rebound tenderness and guarding were absent.

Initial laboratory studies revealed lactic acid 0.6 mmol/L (normal, 0.5-1 mmol/L), albumin 2.6 mg/dL (normal, 3.5-5.5 mg/dL), alanine transaminase 33 IU/L (normal, 10-40 IU/L), aspartate transaminase  19 IU/L (normal, 5-40 IU/L), thyroid-stimulating hormone 1.79 mIU/L (normal, 0.5-4.0 mIU/L), C-reactive protein 42.3 mg/L (normal, <1.0 mg/L). The patient had a negative urine analysis. Routine laboratory examination, including a complete blood count, complete metabolic profile, and pancreatic enzymes were within normal limits. Stool cultures and smear showed rare neutrophils and eosinophils. Cardiac enzymes and hepatitis panel were also negative. The test for tissue transglutaminase immunoglobulin A antibody (tTG-IgA) was negative. Workup for infectious colitis, viral gastroenteritis, inflammatory bowel disease, *Clostridium difficile* infection, irritable bowel syndrome, or malignancy was inconclusive.

A contrast-enhanced computed tomography of the abdomen and pelvis was performed. It demonstrated a large amount of simple-appearing free fluid within the abdomen and mucosal edema throughout the small bowel. The significant amount of free fluid within the abdomen made angioedema of the small bowel, secondary to lisinopril usage, the more likely diagnosis. Colonoscopy with biopsies was normal. The patient did not have any evidence of angioedema of the oropharyngeal area with no associated respiratory symptoms. In the management of our patient, lisinopril was discontinued, after which she started improving clinically with diminution of symptoms. On day two of admission, the patient was discharged from the hospital a day after an unremarkable recovery with education and documentation of this serious ACE inhibitor allergy. At the two-week outpatient follow-up, she had been symptom free and continued to do well. The patient was screened for underlying defects in C1-inhibitor with functional C1-esterase inhibitor and serum C4 level, which were within normal limits. The patient’s hypertension was treated with amlodipine.

## Discussion

ACE inhibitors are a leading cause of drug-induced angioedema in the United States, accounting for 20% to 40% of all the emergency department visits for angioedema annually [1-3]. ACE inhibitor-associated angioedema frequently involves the lips, tongue, face, and upper airway with a well-known female predominance [4]. However, intestinal angioedema secondary to ACE inhibitors is a rare clinical entity. This adverse effect commonly presents within the first four weeks after initiation of therapy and has not shown to be dose related or caused by one particular ACE inhibitor [4-5]. No definitive predisposing factors have been identified, although the current clinical evidence suggests that patients with a history of either hereditary or idiopathic angioedema are at an increased risk for ACE inhibitor-induced angioedema [5].

Recognition of ACE inhibitor-induced small bowel angioedema is difficult and is often delayed. A review of the cases reported in the literature demonstrated the time from the onset of symptoms to the time of diagnosis varies from a few weeks to nine years [6]. Physicians often fail to recognize the association between ACE inhibitors and angioedema owing to the nonspecific clinical presentation and lack of knowledge of this rare adverse reaction. It is also concluded that the continued use of ACE inhibitors despite an episode of angioedema puts patients at much higher risk of recurrent attacks of angioedema [5-6]. Furthermore, a rechallenge with medication may be required to define an adverse drug reaction. In most of the cases with ACE inhibitor-induced visceral angioedema, symptoms recur whenever the culprit drug is restarted. Although life-threatening laryngeal edema following visceral edema due to ACE inhibitors has never been reported [6], it is imperative for clinicians to ensure the safety of patients before initiating a rechallenge.

The differentials of visceral angioedema include bowel wall ischemia, aortic rupture, vasculitis, trauma, malignancy, infections (viz. enteritis), peritonitis, appendicitis, and perforated viscus [5-6]. In patients with bowel wall ischemia, arterial or venous occlusion may be present, and such patients usually have a history of mesenteric insufficiency. Intramural hemorrhage may appear very similar to ACE inhibitor-induced visceral angioedema on contrast-enhanced computed tomography [6]. However, such patients usually have a known bleeding tendency mostly related to anticoagulant use. Small bowel lymphoma can be ruled out as it typically demonstrates a homogeneous density of involved bowel wall and is frequently associated with lymphadenopathy that commonly involves other abdominal structures [7]. Lastly, radiation enteritis may be differentiated from visceral angioedema by the history of irradiation and mural thickening of the bowel wall in a stratified manner in the area of previous irradiation [8]. In our case, these conditions were eliminated with the imaging studies, and the patient had a negative history for probable disorders leading to isolated small bowel angioedema.

Visceral angioedema due to angiotensin receptor blockers (ARBs) has also been reported [9]. ACE inhibitor-induced angioedema is due to the accumulation of bradykinin and its metabolites. However, ARBs produce antihypertensive effects by blocking the angiotensin II type 1 receptor action; hence, bradykinin-related side effects are not expected [9]. The exact pathogenetic mechanism causing visceral angioedema following ARBs is unknown. Recurrent angioedema has been reported to occur in 1.5% of patients after changing from ACE inhibitors to ARBs. Recently, it is suggested that, after an informed consent, ARBs should be considered for use in patients with a history of ACE inhibitor-induced angioedema who have a high therapeutic need for angiotensin inhibition. After discontinuing the ACE inhibitor, waiting for at least four weeks before cautiously starting the ARB is recommended [9].

The management of patients with symptomatic small bowel angioedema from ACE inhibitors is mainly supportive. In a majority of the cases, symptoms usually resolve in 24-48 hours after discontinuation of the culprit medication [8-9]. The efficacy of antihistamines and fresh frozen plasma needs to be evaluated with ACE inhibitor-induced intestinal angioedema in cases refractory to simply stopping the medication [10]. Small bowel angioedema is usually not life threatening. However, it is not uncommon for these patients to receive antibiotics or have a surgical exploration due to diagnostic confusion [1-10]. In patients with bowel wall angioedema who undergo extensive surgical resection of the bowel, multiple morbidities are encountered, which can be entirely avoided by a timely and correct identification of ACE inhibitor-induced angioedema. Occasionally, the diagnosis is made after symptoms return following reinitiation of the ACE inhibitors after hospital discharge. Therefore, a high index of clinical suspicion should be maintained in patients on therapy with ACE inhibitors presenting with gastrointestinal symptoms.

## Conclusions

ACE inhibitor-induced visceral angioedema is under-reported and most often missed, resulting in the waste of hospital resources investigating this clinical diagnosis. In such patients, an appropriate recognition with prompt discontinuation of the drug and appropriate treatment as indicated could prevent an unneeded surgical intervention. Hence, angioedema of the gastrointestinal tract should be considered in all patients taking ACE inhibitors who present with abdominal pain, nausea, vomiting, diarrhea, and/or ascites.

## Appendices

The patient agreed to participate, and the nature and objectives of this study were explained to her. Informed consent was formally obtained. No reference to the patient's identity was made at any stage during data analysis or in the report.
